# Dually Crosslinked Polymer Networks Incorporating Dynamic Covalent Bonds

**DOI:** 10.3390/polym13030396

**Published:** 2021-01-27

**Authors:** Larissa Hammer, Nathan J. Van Zee, Renaud Nicolaÿ

**Affiliations:** Chimie Moléculaire, Macromoléculaire, Matériaux, ESPCI Paris, CNRS, Université PSL, 10 rue Vauquelin, 75005 Paris, France; larissa.hammer@espci.psl.eu (L.H.); nathan.van-zee@espci.psl.eu (N.J.V.Z.)

**Keywords:** covalent adaptable networks, dynamic covalent chemistry, supramolecular chemistry, vitrimers, responsive materials, self-healing materials, shape memory polymers, hydrogels, interpenetrated networks, recyclability

## Abstract

Covalent adaptable networks (CANs) are polymeric networks containing covalent crosslinks that are dynamic under specific conditions. In addition to possessing the malleability of thermoplastics and the dimensional stability of thermosets, CANs exhibit a unique combination of physical properties, including adaptability, self-healing, shape-memory, stimuli-responsiveness, and enhanced recyclability. The physical properties and the service conditions (such as temperature, pH, and humidity) of CANs are defined by the nature of their constituent dynamic covalent bonds (DCBs). In response to the increasing demand for more sophisticated and adaptable materials, the scientific community has identified dual dynamic networks (DDNs) as a promising new class of polymeric materials. By combining two (or more) distinct crosslinkers in one system, a material with tailored thermal, rheological, and mechanical properties can be designed. One remarkable ability of DDNs is their capacity to combine dimensional stability, bond dynamicity, and multi-responsiveness. This review aims to give an overview of the advances in the emerging field of DDNs with a special emphasis on their design, structure-property relationships, and applications. This review illustrates how DDNs offer many prospects that single (dynamic) networks cannot provide and highlights the challenges associated with their synthesis and characterization.

## 1. Introduction

Synthetic organic polymers have found use in an incredibly broad range of applications, spanning single-use packaging materials to specialized medical devices. This versatility is realized at a global scale of production, which is a testament to the capabilities of modern polymer chemistry. In discussing such a diverse body of materials, a practical approach is to categorize polymers as either thermoplastics or thermosets, which essentially distinguishes polymers on the basis of their temperature-dependent flow properties.

Thermoplastics are characterized by a thermal phase transition that results in a viscoelastic fluid. Amorphous thermoplastics behave like brittle solids at temperatures below the glass transition temperature (*T*_g_) and, like viscoelastic liquids, upon heating above the *T*_g_. Semi-crystalline thermoplastics are like ductile solids between the *T*_g_ and the melting transition temperature (*T*_m_), and heating above the T_m_ causes the crystalline domains to melt and the material to flow. Typical commercial amorphous and semi-crystalline thermoplastics have an average molecular weight that is significantly larger than the associated entanglement molecular weight, but these transient physical crosslinks are weak and do not prevent viscous flow. Thus, upon heating to a suitably high temperature (and excluding the impact of side reactions), thermoplastics can be processed and reprocessed by industrial techniques, such as extrusion, injection molding, melt spinning, and 3D-printing. For these reasons, thermoplastics represent about 80% of global polymer consumption [1].

In contrast, thermosets do not flow at any temperature owing to the presence of chemical crosslinks that are permanent and static. When an amorphous thermoset is heated above its *T*_g_, it becomes rubbery and behaves like a viscoelastic solid. Narrow-meshed networks that are glassy solids at service temperature are typically called structural thermosets, while wide-meshed thermosets that exhibit a rubbery elastic behavior at low temperatures (i.e., below 0 °C) are elastomers. In contrast to structural thermosets, elastomers undergo large deformation at service temperatures because of the segmental movement of the chains between the sparsely-distributed crosslinking points. Although thermosets tend to exhibit thermomechanical properties, chemical resistance, and dimensional stability superior to those of their thermoplastic counterparts, they cannot be reprocessed nor recycled because of the permanent and static nature of the crosslinks. 

Beginning over 30 years ago [2], researchers have targeted materials with physical properties that are in between those of thermoplastics and thermosets. One prominent strategy is to introduce exchangeable dynamic bonds into polymer networks. Under certain conditions, the crosslinks are able to shuffle, which permits the topology of the network to respond to external stimuli. In addition to possessing the malleability of thermoplastics and the dimensional stability of thermosets, the resulting materials exhibit a unique combination of physical properties, including adaptability, self-healing, shape-memory, stimuli-responsiveness, and enhanced recyclability.

Dynamic bonds are commonly categorized based on the interaction responsible for the bond’s strength. Non-covalent bonds (NCBs) are those formed by supramolecular interactions such as hydrogen bonding, π–π stacking, dipole-dipole interactions, and van der Waals forces. For the purposes of this discussion, we consider metal–ligand bonds as a form of NCB as well. The strength of NCBs varies widely. For example, the free dissociation energy of neutral hydrogen bonds is on the order of a few kJ·mol^−1^ [3]. The strength of a given metal–ligand bond depends on many factors, such as the identity and oxidation state of the metal, the denticity of the ligand, and the electronic properties of proximal chemical species (e.g., other ligands coordinated to the metal). Metal–ligand bonds can range from tens to hundreds of kJ·mol^−1^ [4]. Most NCBs exhibit a short lifetime, imparting a highly dynamic character to the materials in which they are implemented. The reversible nature of NCBs gives rise to the dynamic character of supramolecular systems [5], which has been exploited to design self-healing and stimuli-responsive materials [6,7,8,9,10,11,12]. However, an undesirable consequence of this design is that these systems tend to creep, thus, limiting the material’s dimensional stability. 

In contrast, dynamic covalent bonds (DCBs) are covalent bonds that can undergo exchange reactions, typically in response to an external stimulus, such as heat, light, and changes in pH. Due to the covalent nature of the interaction, the bond strength of DCBs is significantly larger than that of NCBs, and DCBs have a bond dissociation energy on the order of several 100 kJ·mol^−1^. Polymer networks that incorporate such bonds are called covalent adaptable networks (CANs) [13,14,15]. Thanks to the covalent nature of their interaction, DCBs tend to impart greater creep resistance to dynamically crosslinked materials when compared to NCBs.

The physical properties of dynamic materials that incorporate just one kind of NCB or DCB strongly depend on the nature and kinetics of the bond exchanges within the polymer network. The choice of a single crosslinker restricts the material to a relatively narrow window of properties, which limits the user’s ability to design a material for a desired application. In response to increasing demand for more sophisticated and adaptable materials, the scientific community has identified dual dynamic networks (DDNs) as a promising new class of polymeric materials that combine two (or more) distinct crosslinkers in one system. The different crosslinks can be chosen from a vast variety of static covalent, dynamic covalent, and physical bonds. By combining different crosslinking strategies, a precisely tailored material can be designed. The choice of complementary chemistries can provide easy access to diversified material properties and give rise to synergistic effects. 

A remarkably large number of DDNs have already been reported. DCBs have been exploited in nearly all kinds of polymer materials by now, ranging from bulk materials, like polyurethane (PUR) thermoplastic elastomers (TPEs) and vitrimers to hydrogels. Although it is difficult to form generalized principles for such a diverse field, it is clear that the thoughtful and creative combination of two kinds of crosslinks can lead to materials with unique combinations of properties.

In this review, we aim to give the reader a comprehensive view of the design and properties of dual networks that include at least one type of DCB. In the opening section, a brief overview presents the nature and the properties of DCBs as well as the CANs in which they are integrated. After a brief discussion of the historical context of DDNs, we highlight a variety of materials in the fields of polyurethane elastomers, shape memory materials, vitrimers, hydrogels, and interpenetrated polymer networks.

## 2. Dynamic Covalent Bonds in Polymer Networks

**Figure 1:**
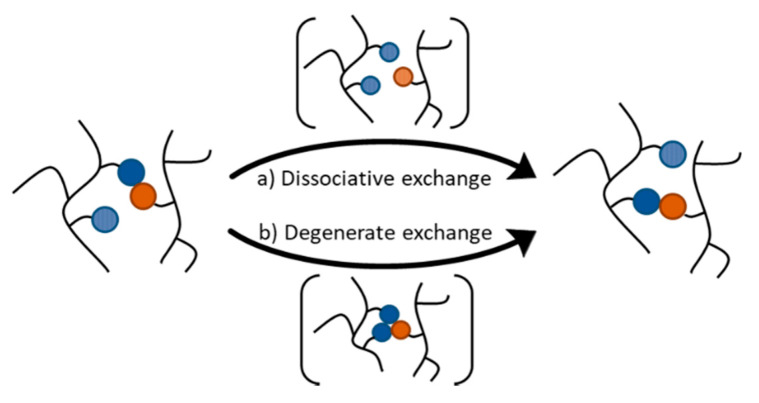
Dynamic bond exchange can proceed via two distinct mechanisms. (**a**) Dissociative exchange: the dynamic bond dissociates before a new one is formed. (**b**) Associative or degenerate exchange: the reaction proceeds via an intermediate (or transition) state in which the entities involved in the exchange are chemically linked together.

The introduction of a sufficiently large number of DCBs into polymer networks yield CANs that have the ability to change their topology by shuffling the DCBs. The first CANs were reported only 20 years ago, consisting of crosslinked polymer materials that contain reversible covalent bonds based on the Diels-Alder (DA) reaction [16]. Subsequent CANs have been developed that extend the scope of dynamic covalent reactions and polymeric matrices. CANs can be divided into two subgroups on the basis of how the exchange reaction proceeds: dissociative and degenerate mechanisms (Figure 1). These different mechanisms have important consequences on how polymer networks behave. A detailed discussion can be found in a recent review by Winne, Leibler, and Du Prez [17], and a brief overview is provided in the present section.

A dissociative exchange mechanism is comprised of a stepwise elimination/addition pathway in which the chemical bond is first broken before it is formed again with another exchange partner. Dissociative exchange reactions take place via an endothermic intermediate step that leads to de-crosslinking and free reactive chain ends. At all times, there is a dynamic equilibrium between associated and dissociated crosslinks. The equilibrium constant and, thus, the crosslinking density are dependent on the temperature. When the equilibrium is significantly shifted to the endothermic side, the connectivity decreases considerably, compromising the network structure. As a result, the viscosity drops significantly and the material behaves more like a thermoplastic. Upon cooling in the absence of side reactions, the crosslinks reform and the physical properties of the network are restored. In solution, materials that solely rely on dissociative exchange reactions undergo a gel-sol transition when the equilibrium is shifted to the dissociated side as a result of the de-crosslinking of the matrix.

In contrast, exchange reactions that follow a degenerate mechanism are characterized by chemical entities on both sides of the equilibrium that are thermodynamically identical. Thus, the reaction equilibrium constant is equal to unity, and it is independent of temperature. Degenerate exchange reactions typically involve an associative addition/elimination pathway. In polymer networks relying on such exchanges, one of the chemical entities involved in the exchange process, which can be a free chain-end, a pendant functional group, or even functional groups within the backbone or crosslinks, adds onto another dynamic covalent linkage. The resulting intermediate (or transition state) is characterized by a higher degree of connectivity. The subsequent fragmentation of this species releases the two chemical partners involved in the degenerate process. In general, the lifetime of the species exhibiting a higher order of connectivity is negligible. Thus, systems proceeding via degenerate exchanges exhibit an essentially constant number of crosslinks at all temperatures. The ability to maintain a constant connectivity while undergoing topological changes results in a gradual viscosity decrease that follows an Arrhenius relationship, which is a property previously found only in vitreous silica. 

Polymer networks containing DCBs that follow a degenerate exchange mechanism are called vitrimers, a term coined by Leibler and co-workers in 2011 [18]. Like other polymer networks, vitrimers have a *T*_g_ that defines the transition between the glassy and rubbery states. Additionally, vitrimers often possess a second characteristic transition temperature called the topology freezing temperature (*T*_v_), which is deriving from the exchange reactions taking place between the DCBs. The *T*_v_ is defined as the temperature at which the melt viscosity is equal to 10^12^ Pa·s [18,19]. However, when the *T*_g_ of the matrix (or the *T*_m_ in the case of semi-crystalline vitrimers) is higher than the theoretical *T*_v_ predicted based on the lifetime of the dynamic bonds, vitrimers do not display a *T*_v_. In these cases, the topology of the network is frozen because of the restricted mobility of the polymer chains associated to the *T*_g_ (or *T*_m_) and not because of the dynamicity of the DCBs present in the network. 

The flow properties of vitrimers are mainly dictated by the rate of exchange of the DCBs, and the evolution of the viscosity as a function of temperature obeys the Arrhenius law. The rate of exchange of the DCBs within vitrimers can be limited by the rate constant of the degenerative process in which case the activation energy (*E*_a_) of the viscous flow is comparable to that of a model exchange reaction between small molecules [18,20]. However, when the concentration of the DCBs is sufficiently low or if the mobility of the DCBs in the matrix is sufficiently restricted, the *E*_a_ of viscous flow may be significantly larger than that of the degenerate exchange between the analogous small molecules [20,21,22].

Although Arrhenius temperature-dependence of the viscosity was initially identified as a hallmark of vitrimers, several CANs with DCBs that follow a dissociative exchange mechanism are reported to also exhibit Arrhenius viscosity profiles [23,24,25,26]. In fact, when the enthalpy for the bond breaking reaction of a dissociative exchange is sufficiently large, the temperature to shift the equilibrium to the de-crosslinked state may exceed the degradation temperature of the material. For such systems, the fraction of dissociated linkages is negligible at processing temperatures, and the network will present an apparent constant crosslinking density despite the dissociative nature of the CAN. 

The insolubility of vitrimers in non-reactive solvents regardless of the temperature [17,27], which is another characteristic that has long been considered to be an intrinsic property of vitrimers, was experimentally contradicted. Recent studies suggest that some vitrimers can partially or completely dissolve depending on the network topology, integrated functionality, dynamics of exchange, and time scale of the dissolution test [22,28]. The dissolution of the vitrimers does not reflect a loss of connectivity but rather the formation of soluble branched and cyclic structures during the reorganization of the network [29,30].

Numerous DCBs have been successfully incorporated into polymer matrices to obtain CANs. Several reviews provide an overview about the state-of-the-art systems in this field [15,17,20,27,31,32,33,34]. We advise readers to take caution when interpreting the use of the term “vitrimer” in the literature. Some systems have been introduced as vitrimers [35], even though they ultimately were found to undergo exchange reactions through a dissociative mechanism [23]. Many of the chemistries found in supramolecular polymer networks and CANs have been exploited and extended to create DDNs. An overview over dynamic covalent bonds found in DDNs is provided in Table 1, and a detailed overview about dual CANs comprising supramolecular bonds can be found in Table 2.

## 3. Origin of Dual Dynamic Networks: Supramolecular Networks

Perhaps the most prominent early example of a DDN is ionomers developed by DuPont in the 1960s [101]. R. W. Rees, working within a team interested in copolymerizing ethylene with functional comonomers, discovered that the sodium salt of ethylene methacrylic acid copolymers has peculiar physical properties. It exhibits unusually high optical clarity, yet it is processable like polyethylene. This early DDN features two different forms of supramolecular crosslinking: the crystallized polyethylene segments of the matrix and the clustering between the sodium carboxylate groups. The combination of these two crosslinks results in a material that is strikingly different compared to virgin polyethylene. Aside from its enhanced optical clarity, the polyethylene ionomers exhibit higher melt strength, higher solid-state toughness, and enhanced oil resistance compared to polyethylene. This finding ultimately led to the commercialization of such materials under the tradename Surlyn, which continues to be sold today.

The group of J.-M. Lehn [46] was the first to combine dynamic covalent chemistry with explicit supramolecular units in dynamic polymers. They designed molecular components that simultaneously generate non-covalent, high-affinity sextuple hydrogen bonds and acylhydrazone links, with both types of bonds featuring dynamic behavior. The resulting DDNs undergo assembly, disassembly, and exchange processes, foreshadowing the great potential of this new class of adaptable materials. Later, the same group combined acylhydrazone chemistry with hydrogen bonds between urea-type groups in siloxane-based linear polymers [60]. The resulting material forms an elastic film that shows self-healing properties. The observed pH-independent time scale of the self-healing process indicates that the healing mechanism predominantly relies on hydrogen bonding. The dynamic exchange of the acylhydrazone units was exploited to perform post-polymerization modification reactions that allowed for the incorporation of new compounds into the polymer [60].

## 4. Polyurethane Elastomers

The most prevalent example of physical networks formed by linear polymers are TPEs. They typically consist of block copolymers that combine two or more incompatible segments that phase separate. One phase is most commonly composed of a high-*T*_g_ or high-*T*_m_ polymer and acts as crosslinking nodules of the continuous, soft, elastomeric phase, which typically consists of a low *T*_g_ amorphous polymer. This composition makes TPEs outstanding materials that not only exhibit the processability of thermoplastics but also the elasticity of vulcanized rubber [102,103,104,105].

One of the most commercially important classes of TPEs is PUR elastomers. In addition to phase separation, intermolecular hydrogen bonding takes place between the urethane groups of the individual chains [106,107]. Several approaches have been reported for upgrading the physical properties of PUR systems through the addition of DCBs. The addition of a second form of dynamic crosslinking is mainly focused on enhancing the self-healing ability of these materials [41,64,65,66]. Other approaches aim to make PUR systems responsive to additional stimuli [57,61,66,108], or impart a new function like damage reporting [57].

Rekondo et al. [41], for example, incorporated dynamic aromatic disulfides crosslinkers into poly(urea-urethane) elastomers, which impart excellent self-healing abilities under ambient conditions. The authors discovered that the self-healing behavior was largely due to the H-bonds in the urea-urethane backbone of the elastomer, as opposed to only the disulfide exchange. This behavior is in contrast to that reported for systems based on aliphatic disulfides, which require an external stimulus and show dynamic behavior though a combination of metathesis reactions and the equilibrium of the thiol-disulfide bonds [109,110]. 

**Figure 2:**
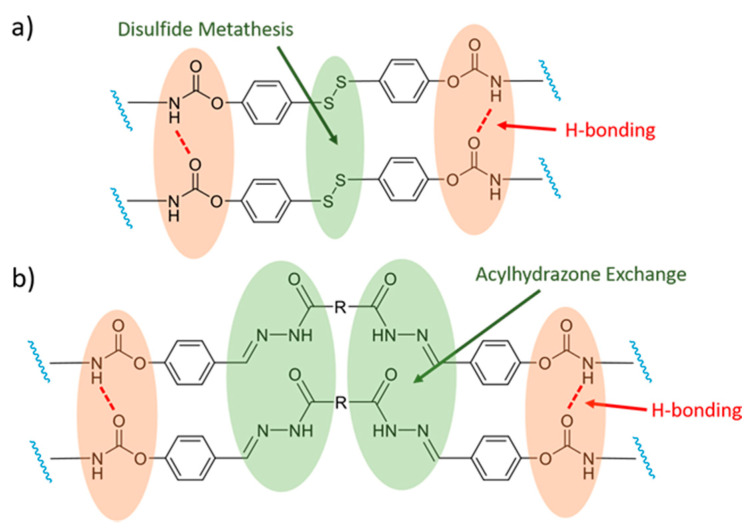
Dual dynamic self-healing PUR elastomers. (**a**) PUR elastomer containing aromatic disulfides [64]. (**b**) PUR elastomer containing acylhydrazone units [108].

A later study by Jian et al. [64] on similar systems (Figure 2a) confirms the important role of H-bonds in the self-healing process. Liu and colleagues [65] extended this concept by introducing a self-healing PUR based on ditelluride bonds, which have a lower dissociation energy than disulfide bonds and, thus, exhibit faster dynamics of exchange. For example, exchanges between ditellurides can take place at room temperature in the absence of light. The H-bond interactions between the polymer chains also played an important auxiliary role in the healing process of this system.

Xu et al. [66] took advantage of the interplay of H-bonding and disulfide dynamic chemistry to design a self-healing PUR. The ability of aliphatic disulfides to undergo dynamic exchange reactions when subjected to sunlight, as well as the mobility of the polymer chains of the colorless transparent matrix, are all pivotal to obtain materials that display light-triggered self-healing properties at room temperature. Furthermore, the hydrogen bonds throughout the PUR matrix are believed to enhance the healing efficiency. When the material presents fractures or cracks, H-bonds favor intimate contact of the damaged surfaces and promote disulfide exchanges between dangling chain ends. Consequently, the healing process results from the rapid closure of the fracture driven by hydrogen bonds, which was followed by a gradual restoration of the mechanical properties thanks to the progressive reformation of the disulfide bonds.

The group of Y. Bai [108] reported the design of two PUR elastomers with one containing dynamic acylhydrazone bonds (Figure 2b) and a second one containing both acylhydrazone and disulfide DCBs. Both kinds of elastomers exhibit self-healing abilities. However, the system containing additional disulfide bonds has a higher healing efficiency. This feature is proposed to result from the ability of the disulfide bonds to exchange faster than the acylhydrazone bonds. Furthermore, the addition of disulfide bonds presumably allows the system to undergo self-healing under neutral conditions at room temperature thanks to the sensitivity of the S–S bond to visible light.

In a distinctly biomimetic approach, the group of P. Sun [36] designed a linear segmented PUR that features H-bonded hard segments that are linked by thermally reversible DA cycloadducts. This design is intended to mimic the hierarchical structures and H-bonding assemblies found in spider silk. The addition of DCBs imparts enhanced mechanical properties to the system, including high strength, stiffness, and toughness, as well as good solvent and heat resistance.

In a recent study, Xu et al. [111] designed a PUR with three complementary dynamic bonds. In addition to the intrinsic hydrogen bonding ability of the urethane groups, the polymer backbone is equipped with thermo-responsive disulfide bonds, and the system was further chemically crosslinked with boronic esters. The goal of this approach was to design a system that shows good mechanical properties, thanks to a certain crosslinking density, while maintaining sufficient mobility of the polymer chains to allow fast and efficient self-healing. This efficiency relies on the different chemical pathways the two exchange reactions offer. Self-healing can be enhanced by the capacity of the boronic esters to undergo hydrolysis, thereby providing a temporal de-crosslinking. At the same time, the crosslinking via the boronic ester endows good mechanical properties and structural stability to the system.

**Figure 3:**
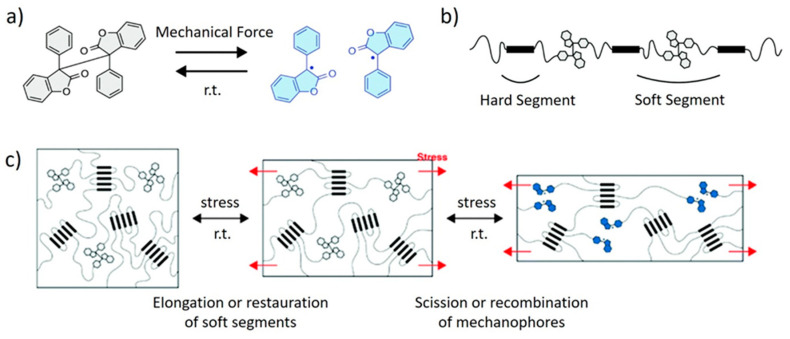
PUR elastomer incorporating diarylbibenzofuranone mechanophores designed by Imato et al. [57]. (**a**) Diarylbibenzofuranone dissociates into stable radicals through C–C scission. (**b**) Structure of the PUR elastomer: the mechanophores are incorporated in the soft segments. (**c**) Mechanism of the uniaxial stretching of the dual PUR elastomer: under the action of stress, the soft segments elongate to full extension, at which point the mechanophores dissociate and emit blue light. Adapted from Reference [57]. Published by The Royal Society of Chemistry 2016.

Imato et al. [57] incorporated diarylbibenzofuranone as a dynamic linker into the soft segments of a PUR elastomer. The mechanism of elongation was thoroughly investigated via tensile tests. Under the action of mechanical stress, the soft segments elongate to full extension, and then the diarylbibenzofuranone mechanophores begin to dissociate into stable radicals via C–C scission (Figure 3). The aggregated hard domains serve as physical crosslinks, and they impart high dimensional stability to the material during this process. When the stress is removed, the extended chains relax and the dissociated radicals recombine. The blue color of the free radicals serves to signal emerging damage.

Another PUR network based on C–C scission, which relied on benzopinacol moieties as DCBs, was introduced by Zhang et al. [61]. Originally used as initiators for free radical polymerization, these aromatic pinacol derivatives form stable radicals upon application of moderate heat or UV irradiation. The dissociation of the benzopinacol units and the rapid recombination of the formed carbon radicals was found to become active at approximately 80 °C, and this behavior allows the bulk material to completely relax stress at this temperature. The network not only shows excellent self-healing and reprocessing characteristics but also high solubility in common solvents when heated above the onset dissociation temperature of the benzopinacol units. These PUR networks can be used as macro-initiators to polymerize methyl methacrylate or styrene in solution. In this process, semi-interpenetrating polymer networks are created, and polymer side chains are grafted onto the network. The grafted polymers are capable of reinitiating a polymerization, providing a reversible-deactivation radical polymerization character to the process. This feature endows this material with attractive polymer engineering possibilities, such as extrinsic self-healing, selective functionalization, or possible re-growing abilities.

## 5. Shape Memory Polymers

Shape memory polymers (SMPs) constitute a class of polymers that intrinsically require a dual crosslinking strategy. As polymeric actuators, SMPs are stimuli-responsive materials able to change their shape to produce motions and forces when subjected to an external trigger [112,113,114,115,116,117]. In general, the polymer is processed by conventional means to obtain its original, permanent shape. In the case of thermo-responsive SMPs, a temporary shape is programmed by heating the material and then shaping it to the desired temporary form, which is then fixed by rapid cooling. Upon a specific trigger signal, the original form can be recovered. The shape recovery takes place when the SMP is heated above a specific transition temperature, most commonly the *T*_g_ or the *T*_m_. 

The permanent shape of many SMPs is fixed by covalent permanent crosslinks. Thus, the shape memory process only works in one direction. As soon as the permanent shape is recovered, the temporary shape is lost and the material must be programmed again. Recently, materials that can alternate between two different programmed shapes without the need to be reprogrammed between cycles have been developed and coined as reversible shape memory polymers. This multi-shape memory effect is made possible by the implementation of additional reversible phase transitions, such as those of aligned liquid crystalline phases [118,119,120,121]. In early exemplifications, the original shape of the material is still defined by the network structure composed of static permanent crosslinks. The reprocessing of such materials is, thus, impossible, limiting the scope of industrial applications and precluding recycling. 

Incorporating DCBs not only provides a means to change the original form of the SMPs but also imparts properties like self-healing, re-processability (reshaping the polymer in its fluidic state), and solid-state plasticity (reshaping polymers permanently without macroscopic melting) while maintaining the mechanical robustness of the materials [122,123,124]. Various dynamic covalent chemistries, such as DA cycloadditions [37,84,85,86,87], photoreversible [2+2] cycloadditions [58,125], imine exchange [49], anhydride exchange [83], transesterification [72,77,94,95,96,125], transcarbamoylation [52,92,93,125], hindered urea bond exchange [92], and disulfide exchange [42,89,90], have been explored to produce adaptable SMPs. Very recently, a comprehensive review about CANs in polymer actuators, such as SMPs and liquid-crystal elastomers (LCEs), has been published by Ji et al. [123].

As an interesting representative example, Xu et al. [91] designed a self-healing polyurethane SMP based on poly(tetramethylene ether glycol), which comprises dynamic disulfide bonds. In this case, the shape memory behavior was utilized to support the self-healing ability of the material. According to the theory of Wool and Conner [126], surface rearrangement and chain diffusion are essential for efficient crack healing in polymeric materials. In the polyurethane SMP, the reversible glass transition of the polyurethane’s soft segments leads to accelerated chain mobility, which significantly promotes the exchange reaction and, thus, the self-healing ability of the material.

DCBs have also been utilized to act as the switch units for programming the temporary shape [42,58,72,88]. Kuang et al. [88] developed a triple shape memory strategy based on reversible and irreversible dual crosslinking of epoxy polymers. The permanent shape of the material was fixed by the static covalent epoxy linkages, while two temporary shapes are programmed by exploiting the glass transition of the epoxy polymer and the presence of thermally reversible DA units. The temporary shape was obtained by heating the material to the temperature at which the retro Diels-Alder (rDA) reaction prevails. The shape was then fixed by cooling down the system and reforming the DA cycloadduct crosslinks. The original form could be recovered by heating to the temperature at which the DA cycloadducts dissociate. The triple shape-memory effect was achieved thanks to the *T*_g_ of the epoxy matrix, which is significantly lower than the temperature of the DA unit dissociation. 

Lendlein et al. [58] prepared a photo-responsive SMP using cinnamic acid and cinnamylidene acetic acid derivatives as switching units. The efficient photoreversible [2+2] cycloaddition of the chromophores made it possible to program to temporary shapes and, subsequently, recover the original shape at ambient temperature by ultraviolet light illumination. Other switches depend on the redox-dependent thiol-disulfide equilibrium [42]. The group of Xie [125] combined thermally induced transesterification and transcarbamoylation with photo-responsive dimerizable cinnamate groups to create a single-component soft robot. The thermo-reversible bonds provide plasticity to the material, while the photo-reversible bonds serve as a shape switch.

**Figure 4:**
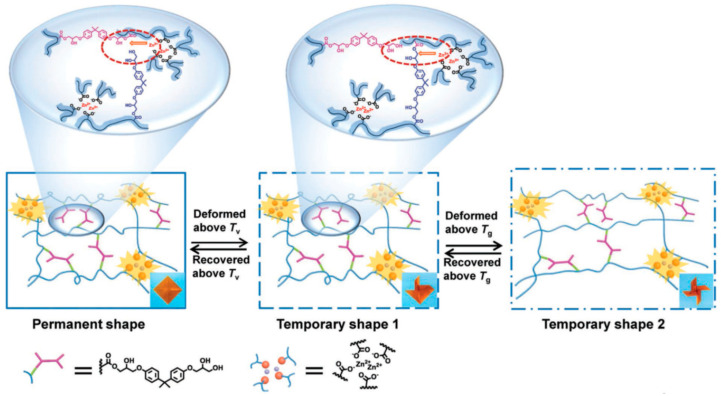
Example of a SMP based on the specific properties of vitrimers. The permanent shape is set by strong ionic interactions. Two temporary shapes can be programmed using the *T*_v_ (temporary shape 1) and the *T*_g_ of the material (temporary shape 2). Adapted from Reference [72]. Published by John Wiley and Sons 2019.

The group of A.-C. Shi [72] reported an innovative SMP that utilizes the properties of vitrimers to create a triple-shape memory material (Figure 4). This system is based on the combination of β-hydroxy ester transesterification and metal-ligand interactions of Zn^2+^ and carboxylate groups. The Zn^2+^ ions not only catalyze the transesterification in vitrimers, as reported by Leibler and co-workers [18], but also act as crosslinking points. The permanent shape is set by the metal-ligand interactions, as they form exceptionally strong physical crosslinks. The authors further exploited the *T*_g_ and *T*_v_ to realize the triple shape memory behavior. The first temporary shape is fixed by the *T*_v_, which is the topology freezing temperature associated to the ester-based DCBs, and the second temporary shape is fixed using the *T*_g_. This system displays high dimensional recovery across programming cycles. Furthermore, the SMP is reprocessable and shows self-healing abilities as a result of transesterification reactions between the hydroxyl and ester groups.

Ji, Terentjev and co-workers [54] used β-hydroxy transesterifications to revolutionize the concept of LCEs, which are actuators that change their shape due to mesogen alignments caused by liquid crystal phase transitions. This transition can be induced by heating above the isotropic transition temperature (*T*_i_) of the liquid crystalline phase. The problem with earlier designs of LCEs was the need to align the liquid crystals before crosslinking, constraining how LCEs can be practically processed because of the risk of inducing disorder to the liquid crystalline phase upon crosslinking. 

**Figure 5:**
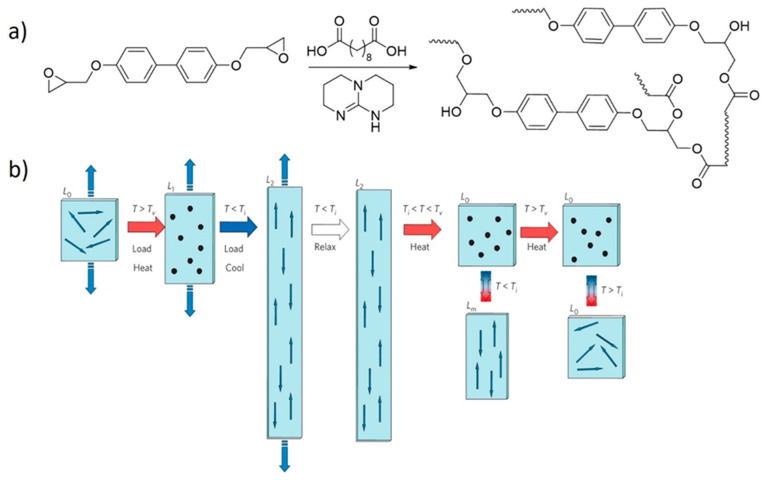
**(a**) Synthesis of an xLCE based on β-hydroxy ester DCBs. (**b**) Uniaxial alignment process of the xLCE. The reshuffling of the network topology, through transesterification reactions, leads to the reversibility of the monodomain alignmet [54].

Terentjev and Ji [54] proposed a new vitrimer-based strategy in which the mesogens are aligned after the formation of the network by heating the system above its *T*_v_ under uniaxial stress (Figure 5). For these systems to behave as actuators, the transition temperatures must be carefully balanced such that the *T*_i_ is well below the *T*_v_. An added benefit of this design is that the material can be reprogrammed by simply heating the system above its *T*_v_. The introduction of DCBs in LCEs, which were coined xLCEs [54], paved the way for simpler fabrication protocols and gave access to complex 3D shapes, making xLCEs eligible for sophisticated high-tech applications like Braille displays [78]. Following this initial report on xLCEs, several studies have adapted and improved the process [77,78,79,80,81,127]. In addition, other dynamic covalent chemistries have been tested, and new xLCEs incorporating disulfide [73,74,75], boronic ester [55], and urethane [76] DCBs, as well as radical-mediated addition-fragmentation chain transfer functionalities [59], have been developed.

## 6. Dual Dynamics in Vitrimers and Related Covalent Adaptable Networks

### 6.1. Vitrimers and CANs with Two Covalent Dynamic Crosslinking Strategies

As highlighted in the context of SMPs and xLCEs, the concept of DDNs has been recently applied to vitrimers. For example, the Zhang group [128] combined transesterification and disulfide metathesis in an epoxy vitrimer. The synergistic combination of these two degenerate exchanges allows the material to undergo topological changes more readily. This feature not only accelerates stress relaxation but also decreases the temperature at which the vitrimer is malleable. As a result, this vitrimer can be reprocessed at 100 °C in 1 h, and the resulting material exhibits complete recovery of mechanical strength. 

Model networks comprising only one type of exchange chemistry, i.e., disulfide metathesis or transesterification, were synthesized for comparing to the corresponding DDN [128]. It must be stated that the respective single dynamic material has the same concentration of disulfide or ester groups as the double network. Therefore, the overall concentration of exchangeable groups is lower in the model single dynamic networks compared to the DDN. At a temperature of 180 °C, the relaxation rate of the dual dynamic vitrimer is approximately 28 times higher than that of the single-disulfide material and 122 times higher than that of the single-ester vitrimer. The synergy of the exchange reactions is also evident in the *T*_v_ of the materials. The experimentally-determined *T*_v_ of the DDN is −6.3 °C, while the *T*_v_ values of the single disulfide and ester vitrimers are 31.0 °C and 71.3 °C, respectively. The malleability at low temperature makes these materials appealing for applications that require self-healing and recyclability. For instance, electronic encapsulation could be a new application of interest. Common vitrimers are not suitable because they tend to exhibit *T*_v_ values that are too large.

**Figure 6:**
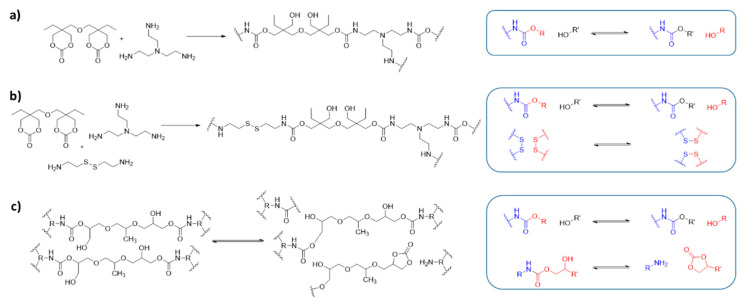
PHU vitrimers. (**a**) PHU vitrimer structure introduced by Dichtel and co-workers [129]. Transcarbamoylation takes place between the urethane groups present in the backbone and the pendant primary hydroxyl groups. (**b**) DDN incorporating hydroxyurethane and disulfide DCBs [131]. (**c**) PHU networks displaying both intermolecular and intramolecular transcarbamoylation as introduced by the Torkelson group [53]. The intramolecular transcarbamoylation leads to the depolymerization of the system.

In 2015, Dichtel and colleagues [129,130] introduced a new vitrimer system based on the transcarbamoylation of polyhydroxyurethane (PHU) (Figure 6a). PHUs are a promising alternative to common PURs due to their nontoxic and ecologically-friendly starting materials. In contrast to conventional PUR thermosets, which are not effectively reprocessed, the PHU vitrimers show dynamic behavior and can be reprocessed without the addition of a catalyst. The material exhibits excellent mechanical properties, although stress relaxation and reprocessing took place only at high temperatures and under pressure (160 °C, 8 h, 4 MPa).

A subsequent study tackled this downside by incorporating a second dynamic mode into the PHU vitrimers in the form of disulfide DCBs (Figure 6b) [131]. Using cystamine as a comonomer, PHU networks with the same excellent thermal stability and mechanical properties as the single dynamic PHU vitrimers are obtained. The introduction of the second DCB results in an increase in dynamicity, making processing possible under milder conditions (150 °C, 30 min). In contrast, a control material containing no disulfide bonds gave only highly inhomogeneous materials in reprocessing experiments, which is consistent with the slower dynamics observed when decreasing the disulfide content in the matrix. However, it should be noted that an increase of the disulfide content is accompanied by a decrease of the crosslinking density, which is a consequence of the different functionality between the bifunctional cystamine and the trifunctional tris(2-aminoethyl)amine (Figure 6b). This change of average crosslinking density is expected to impact both the dynamics and thermo-mechanical properties of the vitrimers.

### 6.2. Vitrimers and CANs with One DCB That Follows Two Different Exchange Mechanisms

Using crosslinks with dual exchange mechanisms represents an elegant approach for achieving the characteristic physical properties of DDNs in a network that contains only one type of DCB. Torkelson and co-workers [53] developed recyclable PHU networks in which both intermolecular and intramolecular transcarbamoylation play an important role (Figure 6c). These networks are obtained using five-membered cyclic carbonates instead of the six-membered cyclic carbonate employed by Dichtel et al. [129,130]. They contain the catalyst 4-dimethylaminopyridine (DMAP). Intramolecular transcarbamoylation restores the monomeric functions, i.e., a cyclic carbonate and an amino group. Even though the intramolecular trans-carbonylation is not a dissociative reaction from a mechanistic point of view, this reaction functionally results in the depolymerization of the network and decrease in its overall connectivity. Both the intramolecular and the intermolecular transcarbamoylation contribute significantly to the network rearrangement with no prevalence of either pathway. This dual process results in a decrease of both the temperature and duration of reprocessing, going from 8 h at 160 °C to 2 h at 140 °C. However, the position of the equilibrium is temperature-dependent because the reversible ring-opening and ring-closing of the cyclic carbonates are not degenerate processes. Thus, depolymerization should be favored above a certain temperature unless the system undergoes degradation.

In 2019, Torkelson and colleagues reported rprocessable polymer networks relying on thiourethane exchange reactions [127]. A dual exchange mechanism was also proposed for this system. Alongside the associative thiol-thiourethane exchange, the system dissociates to its starting products at moderate temperatures. Bowman and co-workers investigated the mechanism of thiourethane exchange as a function of the catalyst [132]. They confirmed the dual mechanism proposed by Torkelson and colleagues, and they showed that it can be altered by the nature of the catalyst.

**Figure 7:**
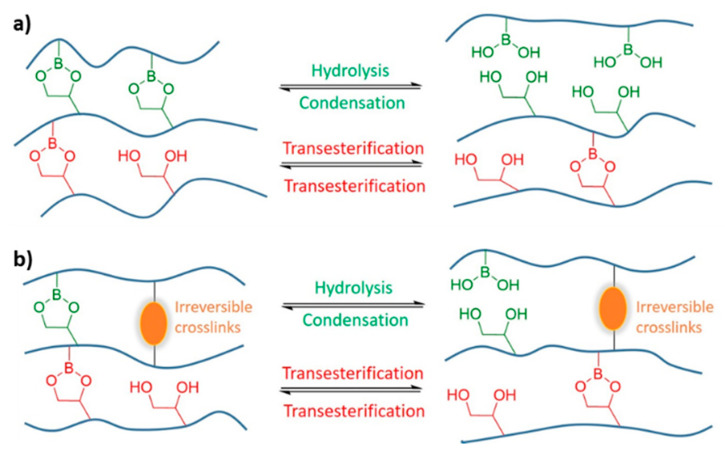
Dual dynamic networks (DDNs) relying on boronic ester dynamic covalent bonds (DCBs) [56]. (**a**) Boronic esters undergo two distinct exchange mechanisms under these conditions. In the presence of moisture, boronic esters undergo reversible hydrolysis and condensation reactions. In the presence of free diols, boronic esters undergo rapid transesterification-based degenerate exchange. (**b**) When the DDN is reinforced with additional static crosslinks, its dimensional stability is enhanced and properties like creep resistance are improved.

Sumerlin and co-workers [56] used boronic esters to further exemplify this strategy. Boronic ester exchange reactions proceed either by a dissociative (i.e., hydrolysis and esterification) or degenerate (i.e., transesterification or metathesis) mechanism depending on the environment (Figure 7a). Due to the hydrolytic sensitivity of boronic esters, dissociation and transesterification are the dominant reactions in humid environments. The transesterification between free diols and boronic esters takes place significantly faster than the metathesis between boronic esters [97,133]. Experiments reveal that the exchange pathway can be easily controlled by simply changing parameters like humidity and diol content, representing another useful tool to tailor material properties.

**Figure 8:**
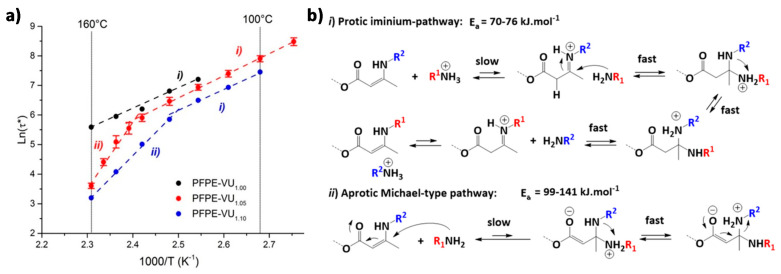
(**a**) Arrhenius plots of fluorinated vinylogous urethane vitrimers [134]. Materials comprising an excess of free amines (PFPE-VU_1.05_, red, and PFPE-VU_1.10_, blue) show two distinct temperature dependencies. The material prepared using a stoichiometric amount of amines (PFPE-VU_1.00_, black) only shows one exchange pathway. (**b**) Two competitive mechanisms: (i) a protic iminium pathway, which prevails at lower temperatures, (ii) an aprotic Michael-type pathway, which requires higher thermal activation and the presence of free amines. Reprinted (adapted) with permission from Reference [134]. Copyright (2018) American Chemical Society.

Guerre, Winne, Du Prez et al. [134] introduced a vitrimer based on vinylogous urethane bonds that comprises two coexisting yet competing bond exchange mechanisms. This study focuses on fluorinated vitrimers relying on vinylogous urethane degenerate exchange [135,136]. While studying the dynamics of these systems through stress relaxation experiments, two different activation energies are observed for the same material depending on the temperature window (Figure 8a). This result differs significantly from the values obtained with model small molecules as well as previous materials based on catalyst-free vinylogous urethane exchange [135]. This behavior is rationalized on the basis of two distinct exchange mechanisms with varying activation barriers. The exchange mechanism that prevails at lower temperature proceeds via a protic iminium species (Figure 8b, part *i*). The dominant pathway at higher temperature involves a direct Michael-type addition of a neutral amine (Figure 8b, part *ii*). In polymers that do not contain free amine species, the Michael-type exchange does not take place. Further investigations revealed that the appearance of the second pathway is related to the network structure of the material. Only materials with larger non-dynamic segments show this kind of behavior, regardless of the presence of fluorinated components.

The approach of using DCBs with dual mechanisms is a promising way to access new materials with varying rheology profiles that can be adjusted to meet specific processing demands. However, it must be emphasized that the variation of some key parameters, such as polymer architecture and composition, can give rise to unexpected behavior even for DCBs seemingly well-known in the community. New reaction pathways may occur that significantly change the behavior of the material, but if they are undetected or if a dual response is misinterpreted as a single response, incorrect conclusions may be drawn. 

### 6.3. Reinforcement by Supramolecular Interactions

Another strategy to manipulate vitrimer properties is to introduce supramolecular bonds that reinforce the network [67,68,70,100,137]. Guan and colleagues [67] introduced H-bonding secondary amide side groups into covalently-crosslinked dynamic polymer networks relying on olefin cross-metathesis as an exchange reaction. The resulting materials show improved mechanical properties, such as elongation and stress at break, compared to materials that do not contain hydrogen bonding units.

**Figure 9:**
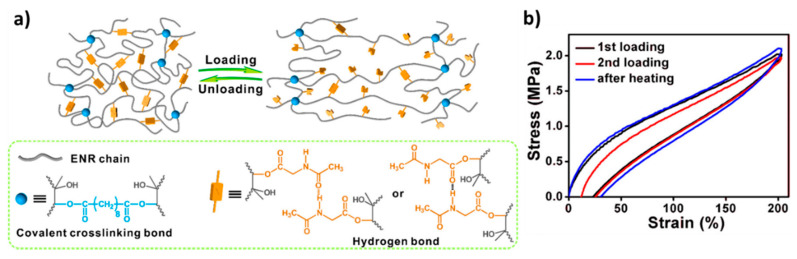
Vitrimer based on dynamic covalent β-hydroxyl ester linkages and supramolecular H-bonds [68]. (**a**) When the material is strained, the H-bonds dissociate. They re-associate when the load is removed. (**b**) Loading-unloading curves illustrating energy dissipation through H-bonds dissociation and reformation. The H-bond reformation is time-dependent and can be accelerated by increasing the temperature. Reprinted (adapted) with permission from Reference [68]. Copyright (2019) American Chemical Society.

Tang, Guo, and colleagues [68] presented an H-bond reinforced vitrimer obtained through concurrent crosslinking and grafting of epoxidized natural rubber (ENR) with sebacic acid and *N*-acetylglycine, respectively (Figure 9). At a high temperature, the rubber network rearranges its topology through degenerate transesterification reactions. The *N*-acetyglycine grafts provide amide functions that form hydrogen bonds. The dynamic breaking and reformation of the H-bonds are able to dissipate energy. Tensile tests demonstrate that the addition of the sacrificial H-bonds leads to a significant improvement to the modulus, ultimate strength, and toughness of the material without sacrificing extensibility.

Loading-unloading experiments exemplify the ability of these elastomers to dissipate a large amount of energy through the breaking and reformation of the sacrificial H-bonds. The reformation of the hydrogen bonds during the loading-unloading experiment is time-dependent and accelerated with an increasing temperature. At low strain rates, due to their finite lifetime, the H-bonds have more time to relax and, thus, contribute less to the modulus of the system than at higher strain rates. Creep and stress relaxation experiments at high temperatures indicate that the network relaxes faster with the increase of the hydrogen units. The grafting of *N*-acetylglycine introduces β-hydroxyl esters into the material, thereby, increasing both the number of hydrogen bonding units and the concentration of DCBs. An increase in the concentration of exchangeable groups leads an increase in the rate of exchange and, thus, a decrease in the lifetime of the dynamic ester bonds at a given temperature.

In a subsequent paper, the same group presented a DDN vitrimer based on epoxidized ENR [137]. Boronic esters are introduced into the system as dynamic crosslinks through the ring-opening of epoxides by a dithiol bis(dioxaborolane) crosslinker. In addition, the system is reinforced with metal-ligand coordination bonds by introducing zinc salts that react with pendant hydroxyl groups along the ENR backbone. At service temperature, the vitrimer with the additional coordination bonds exhibits a higher modulus and stiffness compared to the singly crosslinked network. The DDN still has self-healing capability and re-processability because the metal-ligand bonds are dynamic at higher temperatures. A comparable system based on styrene-butadiene rubber is reinforced in a similar way via the complexation of Zn^2+^ salts and pendant imidazole groups [69].

In a recent paper, Wang et al. [70] incorporated up to three different metal complexes into vitrimers relying on imine exchange. The additional metal-ligand bonds give rise to enhanced creep resistance. The study shows that the extent of creep suppression depends on the stability of the complex formed between the metal ions and the imine-based ligands, revealing another interesting strategy to control material dynamics. 

Another approach to reinforce vitrimers with supramolecular bonds combines epoxy vitrimers with the self-assembly of long alkyl chains [100]. The initial relaxation modulus is increased by the non-covalent interactions of the octyl, dodecyl, or hexadecyl side-chains. The strength of the physical interactions is modulated by selecting the length of the alkyl side chains. 

Looking beyond explicitly-integrated pendant functionality, supramolecular crosslinking in vitrimers can also be derived from interactions originating from the polymer matrix. For example, the crystalline domains of a semi-crystalline vitrimer essentially act as supramolecular crosslinks. This point is analogous to the one made in Section 3 with respect to polyethylene ionomers. A variety of polyethylene vitrimers have already been reported to exhibit novel properties because of the combination of DCBs and the crystallinity of the polyethylene backbone [97,98,138,139,140,141,142,143,144,145]. Along these same lines, microphase separation can also be combined with DCBs to make DDN vitrimers. Sumerlin and colleagues [146] recently exemplified this principle with the synthesis of block copolymer vitrimers, which exhibit distinct viscoelastic properties compared to the statistical copolymer control vitrimer.

### 6.4. Reinforcement by Static Covalent Bonds

To achieve efficient responsiveness, self-healing, and reprocessing at service temperature, bond exchange in a vitrimer needs to operate at an acceptably fast rate. A network possessing DCBs with a short lifetime fulfills these criteria, but the downside is that such materials exhibit continuous and permanent deformation when subjected to mechanical stress. Creep and stress relaxation at service temperatures limit the practical utility of such vitrimers. Numerous elastomeric vitrimers display this undesired behavior at service temperatures [19,22,134,147]. 

This challenge has recently been tackled by complementing dynamic covalent bonds with static covalent crosslinks. Sumerlin and co-workers [56] showed that the incorporation of static covalent crosslinks in a boronic ester-based vitrimer improves the creep resistance such that it is comparable to that of a conventional thermoset (Figure 7b). This DDN still displays self-healing ability, even though bond reformation is slower and less efficient compared to networks containing solely dynamic crosslinks.

Using epoxy-based vitrimers, Torkelson and colleagues [148] further studied the consequences of introducing static crosslinks to vitrimers, trying to find the optimal balance between creep resistance and processability. The authors used Flory-Stockmayer gelation theory to predict the critical content of permanent static crosslinks that a vitrimer should not exceed in order to remain processable. The authors postulate that, for a polymer network to be fully reprocessable, the static crosslinks should be present at a level that is insufficient to form a percolated static network in the material. 

**Figure 10:**
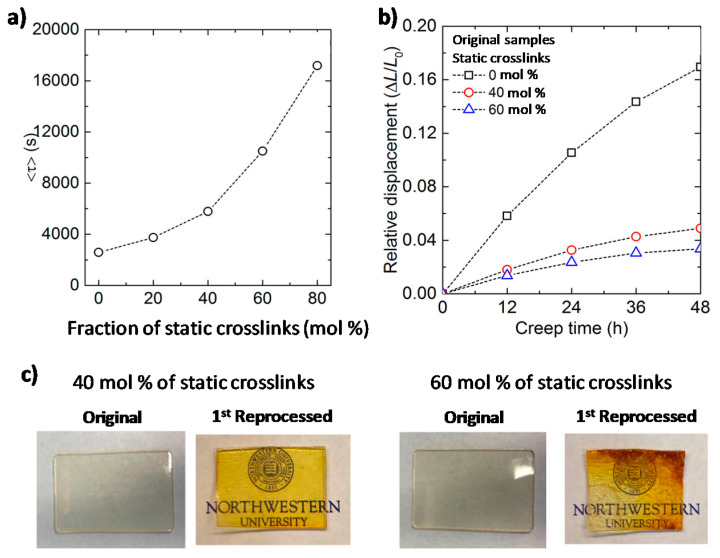
Vitrimers with varying fractions of static crosslinks [148]. (**a**) Average stress relaxation time <τ> as a function of the fraction of static crosslinks. When the proportion of static crosslinks increases, the material requires more time to relax stress. (**b**) Relative displacement as a function of creep time. The incorporation of static crosslinks significantly reduces the displacement. (**c**) Photographic images of samples with 40% and 60% of static crosslinks. The samples comprising 60% of static crosslinks cannot be reprocessed without deterioration of the material. Reprinted (adapted) with permission from Reference [148]. Copyright (2018) American Chemical Society.

In order to experimentally validate this model, thiol-epoxy networks comprising both static and dynamic covalent bonds were prepared. The latter undergo transesterification reactions catalyzed by DMAP. Networks containing either fewer (40%) or more (60%) static crosslinks than the calculated critical value of 50% were prepared. The stress relaxation, creep behavior, and reprocessability of both materials were then compared (Figure 10). Stress relaxation experiments show that increasing the fraction of static bonds in the material results in an increase in the time necessary to relax stress. In addition, the material with 60% static crosslinks shows only limited reprocessability. This sample loses its structural integrity upon heating and deformation, while the sample with 40% static crosslinks retains its shape and shows full recovery of its mechanical properties. In contrast to materials with exclusively dynamic bonds, the network containing 40% of static crosslinks shows 70% less creep. This study convincingly demonstrates that increasing the fraction of static crosslinks in a vitrimer can significantly improve its creep resistance without suppressing its ability to be reprocessed.

Defining a universal criterion regarding the fraction of static crosslinks that can be incorporated into a vitrimer without deteriorating reprocessability is a challenging task. Depending on the structure and functionality of the (macro)molecules used to synthesize the DDN, the weight fraction of the hypothetical static percolated network can vary significantly, as well as the consequential flow properties and processability of the material. The requirements for the flow properties of the polymer melt depend on the desired processing technique (e.g., blow molding, injection molding, extrusion molding, or compression molding). Thus, a dynamic polymer network that contains a secondary percolated static network can be sufficiently processable in certain cases, such as when reprocessing is performed by compression molding after a step of mechanical grinding. Indeed, it has been demonstrated that very strong adhesion can be created between polymer networks incorporating DCBs [149,150,151,152]. 

This point was experimentally confirmed by our group in the context of polybutadiene networks incorporating both static and dynamic dioxaborolane-based crosslinks [22]. For example, a DDN containing static crosslinks such that it has an insoluble fraction of 60 wt % (after the selective cleavage of all the dioxaborolane crosslinks) was efficiently recycled multiple times by compression molding. Moreover, dynamic mechanical analysis (DMA) and mechanical testing indicate that, even after three cycles of recycling (by mechanical grinding and compression molding), the material displays the same crosslinking density and exhibits full recovery of its mechanical properties. As expected based on the observed recyclability, strong adhesion is generated between macroscopic objects made of the dual networks containing both static and dynamic crosslinks. Saed et al. [55] also reported DDNs combining static and dynamic bonds in which the static bonds are incorporated into boronic ester-based liquid crystalline vitrimers to enhance the dimensional stability of the material during actuation.

Besides improving creep resistance, the incorporation of static bonds can be used to tune the dynamics and topological rearrangement of CANs. Nicolaÿ, Matyjaszewski et al. [29] reported one of the first associative CANs, later coined vitrimers, when they designed gels that can be welded in solution thanks to the presence of dynamic covalent trithiocarbonate crosslinks that exchange via an addition-fragmentation mechanism. The introduction of a fraction of static crosslinks to the system permits tuning of the swelling properties of the gels while keeping the overall crosslinking density constant. The higher the amount of dynamic crosslinks, the greater degree to which the gels can reorganize and adapt to the chemical environment. As a result, some of the mechanical stress generated by the diffusion of the solvent within the polymer network is relaxed, and gels presenting a higher swelling ratio are, thus, obtained. 

The Matyjaszewski group also used a combination of static and dynamic crosslinks to develop self-healing coatings [153]. The coatings are constituted of multiarm star polymers that carry dynamic covalent bonds at the periphery, which are used to generate dynamic disulfide crosslinks. This peculiar branched architecture features specific flow properties and distribution of the functionality that enhance the self-healing ability by providing a better accessibility and mobility to the reactive groups. This concept, initially modelled by Matyjaszewski, Balazs, and colleagues through computational simulations [154], was confirmed experimentally by a combination of the atomic force microscopy (AFM) micromachining technique, continuous AFM imaging, and optical microscopy. In this case, static crosslinks are used to precisely control the topology of the polymers, which results in coatings with superior self-healing properties.

Another approach to design DDNs is to integrate dynamic bonds into well-known thermoset systems, such as epoxy resins. This was recently achieved by incorporating disulfide [43,155], imine [50], or vinylogous urethane moieties [156] into common epoxy or diene networks. By integrating DCBs, properties like recyclability and reprocessability can be imparted to typical thermosetting materials.

## 7. Hydrogels

Hydrogels are polymeric networks that absorb and retain large amounts of water. They are generally designed to be biocompatible, stimuli responsive, self-healable, and injectable. Their unique soft rubbery consistency, which is very close to that of living tissues, makes them ideal candidates for a wide range of applications [157,158] especially cell cultures [159,160], cosmetics [161], sensors [162], drug delivery devices [163,164], and tissue engineering [165,166,167]. In recent years, much work has focused on the design of hydrogels based on biopolymers and bio-sourced polymers, like collagen, chitosan, hyaluronic acid, and other polysaccharides in order to develop biocompatible eco-friendly materials [168,169,170]. However, the limited mechanical properties of common hydrogels have precluded their use in applications that call for enhanced mechanical performances. Introducing dual crosslinking is a promising strategy for expanding the scope of applications.

### 7.1. Hydrogels Combining Dynamic Covalent Bonds and Supramolecular Interactions

Sacrificial bonds are commonly employed to improve the mechanical properties of hydrogels. Crosslinks based on NCBs are useful sacrificial bonds because they have short lifetimes under service conditions, which causes the hydrogels to be highly dissipative. Even though the toughness of hydrogels is enhanced, the use of NCBs does not significantly improve the structural stability of the materials because their dynamic nature makes them prone to creep. Most examples of such DDNs rely on either a combination of NCBs and static covalent bonds [171,172,173,174,175], which suppresses the dynamics of the system, or on a combination of different NCBs [176,177,178,179,180], which enhances structural stability only to a limited extent. Combining NCBs with DCBs provides the optimal possibility to improve the structural stability while maintaining adaptability and energy dissipation.

For example, Guo et al. [181] married acylhydrazone chemistry with transient hydrogen bonds to design tough injectable hydrogels. The gels were synthesized by free radical copolymerization of acrylamide and diacetoneacrylamide in the presence of adipic acid dihydrazide (ADH) and polyvinylpyrrolidone. The dihydrazide of the ADH reacts with the ketone groups of the polyamides to form dynamic covalent acylhydrazone bonds, while the polyvinylpyrrolidone forms hydrogen bonds with the amide units of the polyacrylamides, as already demonstrated in physical hydrogels [182]. Previous studies conducted by the authors show that similar hydrogels relying only on acylhydrazone bonds show very poor mechanical properties [183]. The addition of intermolecular hydrogen bonds provides the hydrogels with higher toughness, stretchability, and ductility thanks to synergistic effects. The hydrogels exhibit superior fatigue resistance and self-recovery comparable to that of materials crosslinked with static covalent bonds. These materials feature self-healing properties at ambient conditions without external stimuli as well. 

In addition, the sol-gel transition of these hydrogels is easily controlled through the pH-dependent dissociation of the acylhydrozone bonds. In strongly acidic environments, the acylhydrazone bonds hydrolyze, and the hydrogels turn into injectable viscous solutions. By adding a base, the equilibrium shifts back to the associated form, and tough hydrogels are obtained after *ca*. 2 h. This feature makes it possible to inject the hydrogels to any desired form. The good cytocompatibility and the excellent mechanical strength of these hydrogels are highly desirable attributes for various biomedical applications, such as artificial tissue engineering.

**Figure 11:**
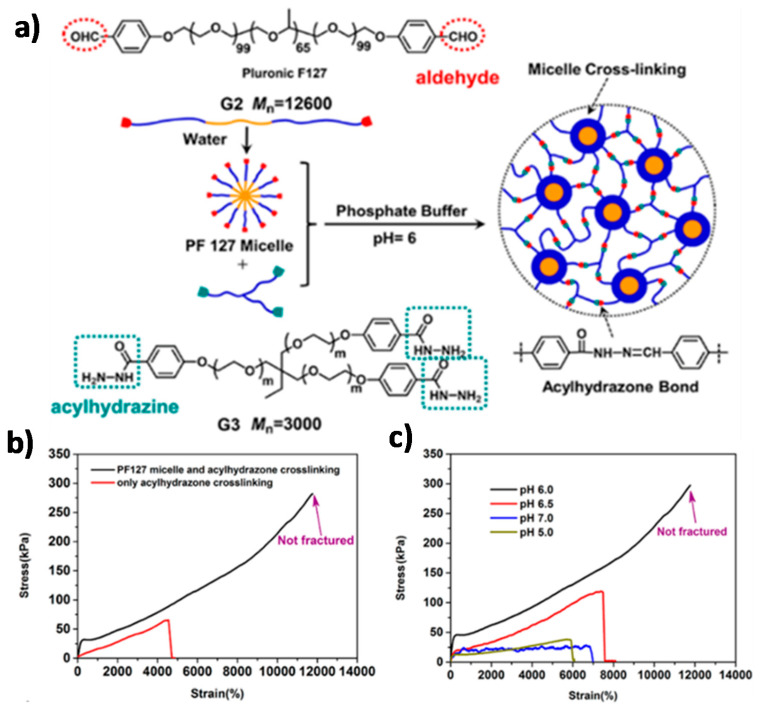
Hydrogels comprising acylhydrazone and micellar crosslinks [99]. (**a**) Synthetic strategy: Homotelechelic aldehyde functionalized triblock co-polymers are crosslinked at a pH = 6 with a three-arm acylhydrazine. (**b**) Uniaxial tensile test of the dual dynamic hydrogel and a hydrogel comprising solely acylhydrazone crosslinks. (**c**) Uniaxial tensile tests of the dual network at different pH values. The gel shows the highest stability at pH = 6.0. Reprinted (adapted) with permission from Reference [99]. Copyright (2017) American Chemical Society.

Chen and co-workers [99] reported a DDN based on the crosslinking of telechelic amphiphilic triblock copolymers. As presented in Figure 11, the aldehyde-terminated polymers form micelles in water, and these structures are crosslinked with a three-arm acylhydrazine linker. Thanks to the combination of the chemical stability of the acylhydrazone DCBs and the dissipative nature of the physical crosslinks, a tough, ultra-stretchable, self-healing hydrogel is obtained. The DDN hydrogel was compared to a similar gel that contained acylhydrazone bonds but no physical interactions, and was also compared to a hydrogel with the same triblock copolymer micellization but with static covalent bonds instead of DCBs. The dual dynamic hydrogel shows superior stretchability and mechanical properties than the reference systems, indicating that both dynamic interactions contribute to the ultra-stretchability and high toughness of the material. The hydrogel exhibits the highest stretchability and mechanical strength at pH 6, which is the pH at which the acylhydrazone bond features the fastest dynamics. At a higher pH, the lifetime of the acylhydrazone bonds increases, while the bonds dissociate at a lower pH, leading to a lower crosslinking density. Cyclic loading-unloading experiments on the different hydrogels indicate that both dynamic interactions contribute to energy dissipation. The dislocation and decomposition of the micelles, in conjunction with the simultaneous chain sliding enabled by the dissociation-reformation equilibria of the acylhydrazone bonds, lead to a pronounced hysteresis in the cyclic experiments, which is proposed as the origin of the material’s excellent mechanical properties.

Polysaccharide-based hydrogels incorporating dynamic oxime crosslinks were also developed by Roh and co-workers [71]. In these systems, the natural ability of alginates to form complexes with divalent cations such as Ca^2+^ was used to create a DDN. The oxime crosslinking permits the formation of viscoelastic hydrogels in microbead or micro-thread geometries. The beads were used to encapsulate and culture 2PK-3 cells. The ability to form biomimetic viscoelastic microenvironments in a defined geometry is a highly desirable feature in biomedical applications.

### 7.2. Hydrogels with Dynamic Covalent Crosslinks of Different Natures

Incorporating two different types of DCBs is an appealing strategy for realizing hydrogels that are strong yet dynamic. For example, one DCB with a short lifetime can be used to impart a dynamic character to the system, and another DCB with a long lifetime can be used as a tunable structural component. Chen and co-workers [38] exemplified this approach by combining acylhydrazone exchange with DA cycloaddition. The cycloadduct-based crosslinks are not dynamic at room temperature, thereby, providing dimensional stability and mechanical strength to the network. The acylhydrazone bonds are highly dynamic under service conditions, conferring self-healing ability to the network. Thanks to the presence of free aldehyde groups involved in the dissociative equilibrium of the acylhydrazone units, the hydrogel shows excellent adhesive properties through the formation of imines with primary amines, rendering the material a promising candidate for tissue engineering applications.

Collins et al. [48] developed a dynamic hydrogel based on oxime groups, which undergo reversible degradation and reformation. The degradation is achieved by transoximination with an excess of both butyraldehyde and trifluoroacetic acid (TFA) at 60 °C. Alternatively, the authors demonstrate a reversible gel-sol transition by treating the oxime-based gel with TFA in dichloromethane (DCM) at room temperature. After rapid degradation of the gel, the TFA (and DCM) evaporates over time, allowing the equilibrium to shift back to network formation. Boronic acid moieties can also be introduced as side-groups. After crosslinking with the polyphenol tannic acid, the resulting material is a dynamic hydrogel with two distinct and orthogonal DCBs.

**Figure 12:**
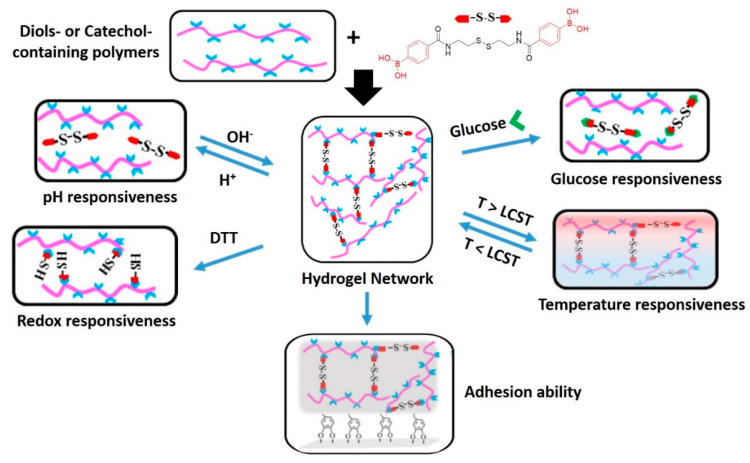
Multi-responsive hydrogel [185]. A pH-, redox-, glucose-, and temperature-responsive hydrogel is obtained through the crosslinking of catechol-functionalized PNIPAM with a disulfide diboronic acid. The resulting hydrogel additionally presents self-healing and adhesion abilities.

The flexibility of design of DDNs has revealed new opportunities for making materials that respond to multiple external stimuli. The group of Zhang [184,185] combined several dynamic covalent chemistries in multi-responsive hydrogels that display quadruple-stimuli sensitiveness (Figure 12). In their studies, diol-containing and catechol-containing polymers were crosslinked with a disulfide diboronic acid crosslinker. The final hydrogels feature not only pH, sugar, redox, and thermal responsiveness, but also enhanced self-healing abilities. The boronic ester bonds are susceptible to hydrolysis in the presence of an acid, but the crosslinks can be reformed under basic conditions. The boronic esters can also be dissociated by the addition of a competitive monofunctional molecule, such as glucose. The disulfide bonds give access to a redox-induced cleavage of the crosslinks. However, the use of glucose or redox reactions lead to irreversible disassembly of the system. Only the pH-dependent hydrolysis-condensation equilibrium of the boronic ester bonds is reversible in this case, which enables the material to exhibit self-healing. When the multi-responsive crosslinker is used to create a network with a catechol-functionalized poly(*N*-isopropylacrylamide) (PNIPAM), the hydrogel shows an additional thermo-responsiveness originating from the lower critical solution temperature of PNIPAM. Furthermore, the hydrogels display advanced adhesive features that stem from the catechol units, which are responsible for the mussel-like adhesive properties.

**Figure 13:**
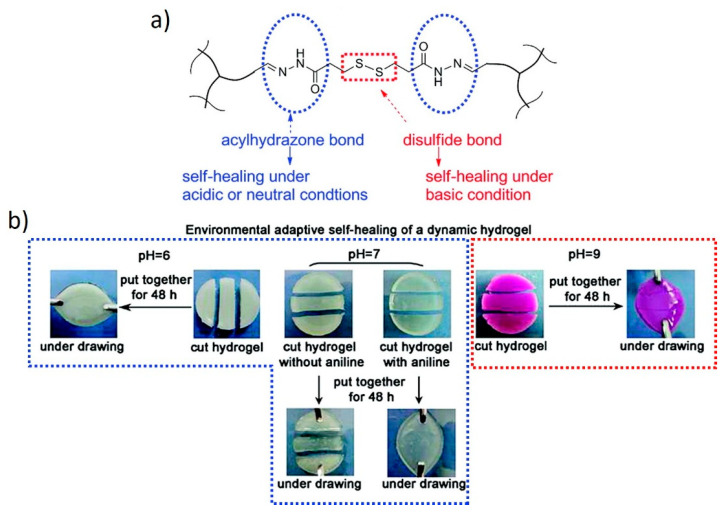
(**a**) Dual dynamic hydrogel based on a combination of acylhydrazone and disulfide dynamic covalent bonds (DCBs) [44]. (**b**) Self-healing of the hydrogel under acidic, basic, and neutral conditions. Reprinted (adapted) with permission from Reference [44]. Copyright (2012) American Chemical Society.

Dual systems can also be used to expand the window of operation within which a stimulus is effective. For example, Deng et al. [44] used two different covalent crosslinkers in responsive hydrogels to extend the self-healing properties to a broader pH spectrum (Figure 13). DCBs, such as acylhydrazone and disulfide bonds, show dynamic behaviors within pH windows that do not overlap. These two chemistries were successfully combined within a single system to obtain hydrogels with self-healing properties under both acidic and basic conditions, thanks to the dynamic nature of acylhydrazone and disulfide bonds, respectively. Self-healing is also possible under neutral conditions in the presence of aniline. Furthermore, these hydrogels undergo pH-induced and redox-induced reversible gel-sol transitions through the selective cleavage of the acylhydrazone or disulfide bonds, respectively.

## 8. Networks Combining Two Different Structures

The combination of two types of dynamic covalent crosslinkers provides the possibility to intimately blend different polymer structures into one material. Many variations are possible, as the polymer structures might differ with respect to the chemical nature of the matrix, crosslinking chemistry, and crosslinking density. In this section, we focus on interpenetrating networks (IPNs) as well as networks that are chemically connected to each other, which we call “combined networks” for the purposes of this discussion.

### 8.1. Interpenetrated Networks

IPNs are a class of polymer alloys in which two or more networks are at least partially intermeshed at a molecular level but not covalently bonded to each other [186,187]. Such a structure is achieved by polymerizing a monomer that has been pre-swollen within an orthogonal polymer network. The objective of this approach is to realize enhanced mechanical features that cannot be achieved with simple blends of the constituent subnetworks (or with either of the individual subnetworks). 

In the early 2000s, the Gong group reported outstanding tough hydrogels featuring mechanical properties that arise from the synergy of two interpenetrated networks: a loosely (statically) crosslinked network interwoven with a densely (statically) crosslinked network. The superior mechanical properties stem from the sacrificial breaking of the highly crosslinked network on extension, which leads to energy dissipation. The work of Gong was taken up by Creton and colleagues [188], who adapted this concept to elastomers. The interplay between both networks leads to a more uniform stress distribution that delays crack propagation. 

A potential drawback of these systems is the irreversible breakage of bonds, and, thus, the significant weakening of the material after the first high-stress loading. A recent approach to address this issue is the addition of dynamic links. Most examples, thus far, feature NCBs [189,190,191,192,193,194]. The introduction of DCBs to IPNs is a new topic of research, and the few examples that exist nicely exemplify the potential of dynamic covalent chemistry, both in terms of versatility and control of the performances. 

The combination of reversible acylhydrazone and boronic ester crosslinks allowed Yang, Zhang, and colleagues [47] to prepare a multi-functional, self-healing, injectable IPN. The network was synthesized from four functional poly (*N*, *N*-dimethylacrylamide) prepolymers equipped with either aldehyde, hydrazone, boronic acid, or catechol side-groups. When these prepolymers are mixed together, the aldehyde groups selectively react with the hydrazide to form acylhydrazide linkages, while the boronic acid and the catechol moieties form boronic esters. The orthogonality of the reactions leads to the formation of two interlocking yet independent subnetworks. The innovative synthetic strategy of mixing components together that feature a fast-gelation behavior make this hydrogel an ideal candidate for 3D-printing solutions.

The two bonds display dynamic behavior in different pH ranges. The acylhydazone bonds are stable in mildly acidic environments but dissociate under basic conditions, whereas the boronic ester bonds only form under basic conditions. As a result, the hydrogel self-repairs under both acidic and basic conditions, but it shows chemical stability under neutral conditions. Thus, by varying the pH to acidic (or basic) conditions, one subnetwork undergoes a gel-sol transition while the other one maintains its gel state. This reversible transition from an IPN to a network penetrated by a linear or branched polymer allows for the pH-dependent tuning of the mechanical properties and average pore size of the hydrogel.

**Figure 14:**
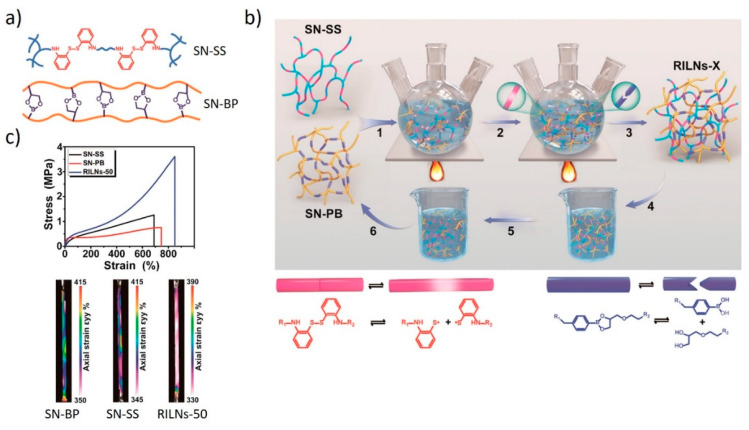
Dual dynamic bulk IPN comprising disulfide and boronic ester DCBs [45]. (**a**) Structure of the disulfide (SN-SS) and the boronic ester (SN-PB) single networks. (**b**) Synthesis of the IPN. The single networks are mixed (1) and the dynamic bonds dissociate under the influence of heat and moisture (2) and interlock (3) when heating is stopped and moisture removed. The process is reversible through the specific hydrolysis of the boronic ester crosslinks in the presence of water (4 and 5). The individual networks can, thus, be separated (6). (**c**) Tensile properties of the single networks and the IPN. Due to the synergistic effects of the two interwoven networks, the strain is better distributed in the IPN, which leads to improved mechanical properties. Reprinted (adapted) with permission from Reference [45]. Copyright (2020) American Chemical Society.

Another approach for combining two DCBs in an IPN was reported by Rong, Zhang, and co-workers [45]. A bulk IPN was prepared from two single PUR elastomer networks, with one comprising boronic esters and the other comprising disulfides (Figure 14). These materials are used to make a reversibly interlocking network (RILN) through the simultaneous and orthogonal exchange of the boronic esters and disulfides present in the respective single networks. The exchange of the DCBs over time leads to the formation of an interlocking topology architecture. In combination with the formation of intermolecular hydrogen bonds between the urethane groups of the two polymer networks, this dynamic interlocking process resulted in the formation of a homogenous material. The RILN shows superior non-linear mechanical properties when compared to the individual subnetworks. This improvement is explained by synergistic effects between structures that lead to improved load bearing capability and more homogeneous stress distribution. 

The two exchange reactions can be triggered selectively by orthogonal external stimuli. The boronic ester bonds are thermally stable, but they can be cleaved by adding water and reformed when the latter is removed. In contrast, the disulfide bonds are hydrolytically stable and are cleaved homolytically at elevated temperatures. Hence, through the selective hydrolysis of the boronic esters, the IPN can be disassembled and the original single networks can be recovered.

Inspired by the groundbreaking work of the Gong group [195,196,197,198], Konkolewicz and co-workers [39,62,63] designed DDN IPNs in which the sacrificial bonds are H-bonds formed between pendant 2-ureido-4[1H]-pyrimidinone (UPy) moieties. The second crosslinker relies on long lifetime DA cycloadducts. Thanks to the high dynamicity of the sacrificial bonds, the network of covalent bonds is conserved, postponing the weakening of the material. The combination of dynamic bonds with two significantly different lifetimes allows for the preparation of IPNs that exhibit improved stress at break and fracture toughness, as well as self-healing and reprocessability, while presenting good creep resistance at room temperature.

**Figure 15:**
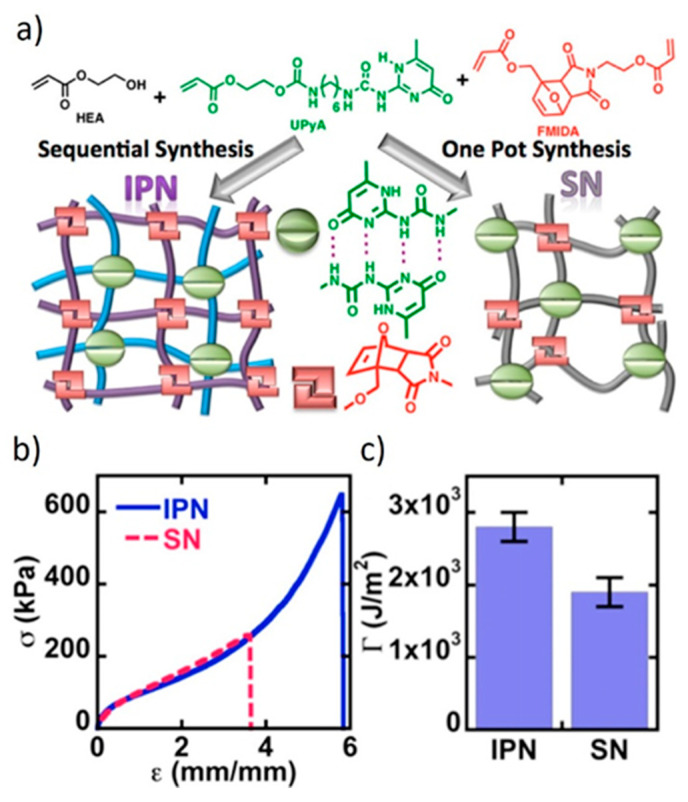
DDN incorporating DA cycloadducts and UPy units as dynamic crosslinkers [39]. (**a**) Synthesis of a dual dynamic interpenetrating networks (IPN) through sequential polymerization. Each subnetwork contains solely one type of crosslinker (left). Synthesis of an SN containing both types of crosslinkers via a single step copolymerization (right). (**b**) Tensile properties of the IPN and the SN. (**c**) Fracture energies of both networks. The IPN generally displays superior mechanical properties than the SN. Reprinted (adapted) with permission from Reference [39]. Copyright (2017) American Chemical Society.

In one of their studies, Konkolewicz and colleagues [39] compared the properties of an IPN and a single network (SN), which are both crosslinked with UPy units and DA cycloadducts (Figure 15). The IPN features superior mechanical properties, as it exhibits improved peak stress, strain at break, and fracture energy. The IPN also displays strain hardening behavior. The separation of both crosslinkers facilitates the energy dissipation, which is also evident in loading-unloading experiments. The SN shows less creep and longer stress-relaxation. The ability of the IPN to partially relax stress faster at room temperature and to deform faster during creep experiments likely reflects the capacity of the subnetworks to move independently in combination with the fast dynamics of the UPy subnetwork. However, both networks show complete creep recovery, which is induced by the DA bonds that display a static behavior under the conditions of the creep experiment.

With respect to self-healing and malleability, the IPN also outperforms the SN. The decoupling of the networks gives the IPN more degrees of freedom, at least with respect to the subnetwork crosslinked with short lifetime dynamic bonds, thus, facilitating the dynamic bond exchange. The studies of the Konkolewicz group are a striking example on how molecular architecture can influence the macroscopic properties of a DDN.

### 8.2. Combined Networks

In contrast to IPNs, so-called combined networks are constituted of two different network structures that are not necessarily interlaced but are chemically connected. For example, Tao and colleagues used this strategy to design a multi-responsive hydrogel consisting of two different networks crosslinked with dynamic imine and boronic ester moieties [51]. Using the Ugi reaction, a tetrafunctional poly(ethylene glycol) (PEG) with a benzaldehyde group and phenylboronic acid group at each chain-end was prepared from a dicarboxy-homotelechelic PEG. This tetrafunctional PEG was used to generate a combined network by selective crosslinking of the benzaldehyde moieties with glycol chitosan and crosslinking the phenyl boronic acid groups with poly(vinyl alcohol). The combined network shows superior self-healing abilities and good mechanical strength. The synergy between the different network structures also provides the material with enhanced muco-adhesive abilities. Furthermore, the hydrogel shows excellent biocompatibility, and it was injectable thanks to the short lifetime of the dynamic crosslinks. This DDN hydrogel was, thus, used as a drug-delivery carrier to transport the anti-cancer drug doxorubicin to a tumor. The slow and gradual release of the drug associated with the hydrolysis of the crosslinkers shows significant advantages over the direct delivery to the tumor.

**Figure 16:**
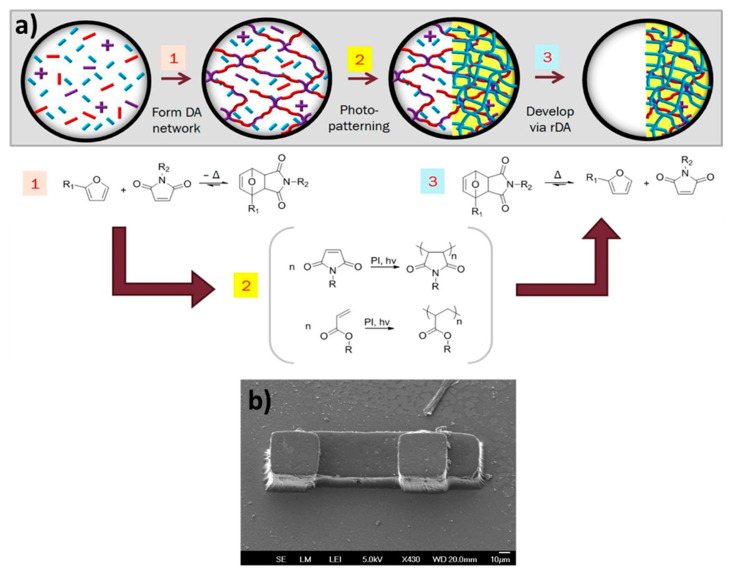
Dual cure system based on DA cycloadducts and static covalent bonds [40]. (**a**) In the first step, the support network is formed via DA cycloaddition (1). During the photopatterning, a static permanent network is created within the DA network (2). In the development step, the support network is removed through thermo-activated rDA depolymerization (3). (**b**) The scanning electron microscopy (SEM) image of two-layer structures created with the dual cure photoresist process. Reprinted (adapted) with permission from Reference [40]. Copyright (2014) American Chemical Society.

The Bowman group [40] reported a unique combination of two different polymer networks relying on dynamic and static covalent crosslinks to create an adaptable solid-state photoresist via stereolithography (Figure 16). In a typical stereolithography process, a liquid monomer is selectively photo-irradiated to generate an insoluble chemical network where the resin is exposed to light. The unreacted resin is later removed during the development process, typically through dissolution in suitable solvents. However, the creation of 3D objects often requires a physical support to hold the structures during their formation to prevent collapse, sedimentation, or loss of resolution. 

To address this challenge, Bowman and colleagues developed a double network photoresist, which is synthesized via a dual-cure process. In the first step, a scaffold network composed of reversible DA adducts is synthesized. The “‘click” nature of this reaction allows for an efficient synthesis of the support network, while the rDA reaction at an elevated temperature imparts thermo-reversibility to the network. The second step of the curing process consists of the photopolymerization of acrylate monomers present in the supporting network. This strategy allows for the spatial and temporal control over the formation of the second network within the first one. Using a multifunctional crosslinker bearing furan and acrylate functions allows for the covalent connection between the two networks. In the development step, the DA network not involved in the second curing step (i.e., the material that had not been exposed to light irradiation) is removed by thermal depolymerization. The use of an easily-removable scaffold network based on a CAN offers new possibilities for layer-by-layer stereolithography applications, as it addresses the challenging removal of the support material from complex or microscale objects.

## 9. Conclusions and Outlook

This review provides an overview of the development of a new class of CANs that incorporates multiple crosslinkers featuring different degrees of dynamicity. The elaboration of new crosslinking strategies in recent years, combined with the demand for more sophisticated materials, has motivated the design of DDNs, resulting in materials that feature unprecedented properties. By combining DCBs with static covalent, supramolecular, and other kinds of dynamic covalent bonds, materials can exhibit significant advantages over those relying solely on just one kind of dynamic crosslinking. Whereas, in singly crosslinked materials, there are always trade-offs between structural stability, dynamicity, adaptability, and responsiveness, DDNs offer the possibility to combine seemingly contradictory features. The diverse examples presented in this review illustrate how DDNs have already become an indispensable strategy in polymer science with applications already ranging from actuating robots [125] to adaptable bio-materials [184,185].

As the field of CANs continues to mature, more dynamic crosslinkers will be combined in increasingly diverse matrices. A key challenge in this context is that it is not trivial a priori to identify the adequate combination of crosslinkers to realize a targeted set of properties. Moreover, simple and scalable synthetic strategies will need to be developed to allow flexibility in combining different kinds of dynamic crosslinkers. The characterization of DDNs must also be advanced to address the difficulty in differentiating between the individual responses of the different dynamic modes [134]. Although many DDNs are directly compared to their respective single networks, a precise and meaningful comparison is not always achievable in this way. Minor changes in the chemical nature, topology, crosslinking density, and functionality can strongly influence how a given dynamic bond behaves in a given DDN. The productive future of this field will rely on the simultaneous advancement of modeling, synthesis, characterization, dynamics, and material development. Many intriguing DDNs have already been reported, but even more fascinating paths forward remain open for exploration.

## Figures and Tables

**Table 1:** Dynamic covalent bonds used in dual dynamic networks (DDNs): Dynamic bonds, stimuli triggering the exchange, polymeric systems developed (with a representative example from the literature), and imparted functions (PUR = polyurethane, SMP = shape-memory polymer, IPN = interpenetrated network).

Dynamic Bonds	Triggers	Polymeric Systems	Imparted Functions
Diels-Alder cycloadduct	*T*	PURs [36], SMPs [37], hydrogels [38], IPNs [39], combined networks [40]	reshapeability, enhanced mechanical properties, self-healing ability, facilitated synthesis, structural stability, shape memory
Disulfide	redox, light, *T*, pH	PURs [41], SMPs [42], vitrimers [43], hydrogels [44], IPNs [45]	reshapeability, self-healing ability, enhanced mechanical properties, responsiveness, facilitated synthesis, shape memory
Acylhydrazone	pH	supramolecular polymers [46], hydrogels [44], IPNs [47]	reshapeability, self-healing ability, responsiveness, facilitated synthesis, injectability
Oxime	*T*, pH, addition of molecules	hydrogels [48]	structural stability, self-healing ability, enhanced mechanical properties
Imine	*T*, pH, addition of molecules	SMPs [49], vitrimers [50], combined networks [51]	reshapeability, self-healing ability, enhanced mechanical properties, facilitated synthesis
Urethane/Urea	*T*, catalysts	SMPs [52], vitrimers [53]	reshapeability, enhanced mechanical properties, facilitated synthesis
Ester	*T*, catalysts	SMPs [54], vitrimers [54]	reshapeability, facilitated synthesis, shape memory
Boronic ester	*T*, pH, addition of molecules	SMPs [55], vitrimers [56], hydrogels [48], IPNs [47], combined networks [51]	reshapeability, self-healing ability, enhanced mechanical properties, responsiveness, facilitated synthesis
C–C (scission)	mechanical force, *T*	PURs [57]	self-healing ability, responsiveness, enhanced mechanical properties
Cycloadduct of cinnamic acid derivatives	light	SMPs [58]	shape memory
Allyl sulfide and trithiocarbonate	*T*, radicals, catalysts	SMPs [59], vitrimers [29]	reshapeability, self-healing ability, facilitated synthesis

**Table 2:** Supramolecular systems found in dual dynamic networks (DDNs): Physical interactions or systems, combined dynamic covalent bonds (DCBs), polymeric systems developed, and imparted functions.

Physical Interactions	Combined DCBs	Polymeric Systems	Imparted Functions
Hydrogen bonds	acylhydrazone [46,60], C–C [57,61], DA cyclo-adduct [36,39,62,63], disulfide [41,64,65,66], imine [49], olefin [67], ester [68]	supramolecular polymers, PURs, SMPs, vitrimers, hydrogels, IPNs	self-healing ability, enhanced mechanical properties
Metal-ligand coordination	boronic ester [69], imine [70], oxime [71], ester [72]	SMPs, vitrimers, hydrogels	structural stability, enhanced mechanical properties, creep, solvent, and acid resistance
Liquid crystals	allyl sulfide [59], boronic ester [55], disulfide [73,74,75], carbamate [76], ester [54,77,78,79,80,81,82]	SMPs	shape memory
Microphase separation	anhydride [83], DA cycloadduct [37,84,85,86,87,88], imine [49], disulfide [89,90,91], urethane/urea [52,92,93], ester [72,77,94,95,96], boronic esters [97,98]	SMPs, vitrimers	shape memory, self-healing ability
Self-assemblies	acylhydrazone [99], ester [100]	vitrimers, hydrogels	self-healing ability, enhanced mechanical properties

## Data Availability

The data presented in this study are available on request from the corresponding author.
